# Molecular Diversity of Mytilin-Like Defense Peptides in Mytilidae (Mollusca, Bivalvia)

**DOI:** 10.3390/antibiotics9010037

**Published:** 2020-01-19

**Authors:** Samuele Greco, Marco Gerdol, Paolo Edomi, Alberto Pallavicini

**Affiliations:** 1 Department of Life Sciences, University of Trieste, 34127 Trieste, Italy; SAMUELE.GRECO@phd.units.it (S.G.); edomi@units.it (P.E.); pallavic@units.it (A.P.); 2 National Institute of Oceanography and Applied Geophysics, 34151 Trieste, Italy; 3 Anton Dohrn Zoological Station, 80121 Naples, Italy

**Keywords:** bivalves, mussels, antimicrobial peptides, innate immunity, defensins

## Abstract

The CS-αβ architecture is a structural scaffold shared by a high number of small, cationic, cysteine-rich defense peptides, found in nearly all the major branches of the tree of life. Although several CS-αβ peptides involved in innate immune response have been described so far in bivalve mollusks, a clear-cut definition of their molecular diversity is still lacking, leaving the evolutionary relationship among defensins, mytilins, myticins and other structurally similar antimicrobial peptides still unclear. In this study, we performed a comprehensive bioinformatic screening of the genomes and transcriptomes available for marine mussels (Mytilida), redefining the distribution of mytilin-like CS-αβ peptides, which in spite of limited primary sequence similarity maintain in all cases a well-conserved backbone, stabilized by four disulfide bonds. Variations in the size of the alpha-helix and the two antiparallel beta strand region, as well as the positioning of the cysteine residues involved in the formation of the C1–C5 disulfide bond might allow a certain degree of structural flexibility, whose functional implications remain to be investigated. The identification of mytilins in *Trichomya* and *Perna* spp. revealed that many additional CS-αβ AMPs remain to be formally described and functionally characterized in Mytilidae, and suggest that a more robust scheme should be used for the future classification of such peptides with respect with their evolutionary origin.

## 1. Introduction

Antimicrobial peptides (AMPs) are fundamental effector molecules of the innate immune system, which are present in nearly all living organisms, serving as a first line of defense against pathogens in the humoral immune response. AMPs have acquired a role of primary importance along the evolution of invertebrate metazoans, which lack an immunoglobulin-based adaptive immune system and have developed a larger and more diversified repertoire of innate immune molecules compared with vertebrates. Some filter-feeding aquatic organisms, such as bivalve mollusks, seem to have developed a remarkably rich arsenal of AMPs and other defense peptides, which might be interpreted as a strategy to efficiently manage bacterial infections from opportunist pathogens found in the water column [1].

The history of AMP research in mollusks has been always linked with marine mussels (*Mytilus* spp.), starting from the identification of several short cysteine-rich peptides from active hemolymph fractions of *Mytilus galloprovincialis* and *Mytilus edulis* in the mid-90s [2,3]. Over the following years, several experimental studies have clarified many aspects concerning the biological function, the regulation and the spectrum of activity of these antimicrobial molecules. Early reports revealed that most mussel AMPs are produced as inactive precursors and stored in granules within hemocytes, the main circulating cells with immune functions (i.e., phagocytosis, encapsulation of invading pathogens and production of cytotoxic molecules) in mollusks [4]. Albeit with different strength and specificity, all mussel AMPs were found to be active against Gram-positive and Gram-negative bacteria and, in some cases, also against viruses, fungi and protozoans [2,3,4,5,6]. However, recent studies have also evidenced that some mussel AMPs, most notably myticins, also display cytokine-like properties [7].

The sequence diversity of the AMPs isolated from mussel hemolymph made immediately clear that they belonged to distantly related—or even completely unrelated—groups. Indeed, while some of them were similar to previously described insect defensins [8,9], others did not bear significant homology with any other known sequence, and were therefore assigned different names. In spite of a lack of primary sequence similarity, three of these AMP families (i.e., defensins, mytilins and myticins) were characterized by the presence of eight cysteine residues, involved in the formation of four disulfide bonds, which stabilize the mature peptide in a compact, cationic and amphipathic structure [10].

Moreover, the 3D structures of the mussel defensins and mytilins, experimentally determined by NMR, revealed that these two AMP classes adopted a similar cysteine-stabilized alpha helix/beta-sheet (CS-αβ) three-dimensional folding, comprising a single alpha helix and two anti parallel beta strands, with the four expected disulfide bonds engaged in a C1-C5, C2-C6, C3-C7 and C4-C8 array [11,12]. Although the structure of myticins has not been experimentally determined yet, structural modeling approaches suggests that these AMPs may also bear a similar CS-αβ fold, stabilized by the same disulfide array described above [13].

Besides mussel AMPs, the CS-αβ motif, stabilized by either three or four disulfide bonds, is found in several other peptides with toxic and defense functions, which display a widespread distribution in nature and little to no primary sequence homology with each other. The remarkable structural similarity among CS-αβ peptides from distantly related taxa led some authors to propose a common origin from an ancestral prototypical sequence [14]. In mussels, this hypothesis is further supported by the similar gene architecture of all known CS-αβ AMPs, as well as by the similar tripartite organization of the precursor peptides [10].

In the present manuscript, we focused our attention on mytilins, one out of the three mussel hemocyte-specific CS-αβ AMP families described so far. Like other mussel AMPs, mytilins show a more potent anti-bacterial activity against Gram+ bacteria (displaying a MIC as low as 0.6 µM against some strains) compared with Gram– bacteria, which are generally only affected at significantly higher concentrations. Mytilins have been identified both in granular hemocytes, recruited at infection sites, and in plasma, suggesting that these AMPs have a primary role in killing invading bacteria upon phagocytosis, and a secondary systemic role exerted by the fraction of the peptide released in the plasma [15,16].

**Figure 1:**
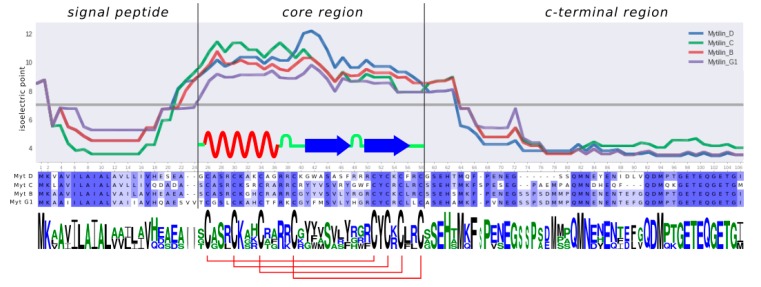
Secondary structure, isoelectric point distribution, sequence conservation logo and disulfide bridge array for mytilin B, C, D and G1 of *Mytilus galloprovincialis*. The isoelectric point plot was generated with a sliding window size of 15 amino acids. The secondary structure of the mature peptide region reports experimental data obtained for mytilin B (PDB: 2EEM).

Mytilin genes comprise four exons and three introns [17] and encode precursor peptides whose structure can be summarized as follows (Figure 1): (i) a N-terminal signal peptide (SP), which targets the precursor to the secretory pathway; (ii) a core region corresponding to the mature, cysteine-rich, cationic and biologically active peptide and (iii) an anionic C-terminal extension, which may facilitate the 3D folding of the mature peptide region and neutralize the positive charge of the mature peptide until its release from storage granules.

Four major types of mytilins have been described in *M. galloprovincialis*, i.e., mytilin B (and its allelic variant F), C, D and G1, which are all characterized by a C-X (3)-C-X (3)-C-X (4)-C-X (11)-C-X-C-X-C-X (2)-C cysteine array and display a modest level of inter-individual sequence variability compared with other mussel AMPs [18]. More recently, three additional sequences have been added to the family: mytilin K and N display some unique features, such as a longer loop between C1 and C2, paired with the unusual position of C5. In spite of the presence of a canonical cysteine array, the primary sequence of the third new mytilin, named pseudomytilin, was largely divergent, in particular in the C-terminal extension region [10].

Although mytilins are most certainly present in the other inter-fertile species of the *Mytilus edulis* species complex [3,19,20], as well as in other species of the same genus, such as a *Mytilus coruscus* [21,22], to date they have never been reported in other species of the order Mytilida. Nevertheless, the evolutionary relatedness of all marine mussels and their adaptation to similar coastal environments suggests that other unexplored species may harbor a similarly complex complement of AMPs, which may also include mytilin-like peptides.

The application of next generation sequencing to the Mytilidae [23,24], in particular thanks to the feasibility of whole genome sequencing approaches [25,26,27], now offers new opportunities for the discovery of novel AMP sequences in species that have been so far subjected to limited scientific attention. In the present work we describe for the first time the presence of mytilin-like sequences in two new genera, i.e., *Perna* Philipsson 1788 and *Trichomya* Ihering 1900, which implies the possibility that related sequences might be found in other phylogenetically related species in the near future. Moreover, we discuss the evolutionary relationship between the newly identified and previously known sequences, report the 3D structural prediction of the newly identified sequences obtained by threading and propose a hidden Markov model (HMM) for the future identification of mytilin-like peptides, which might also aid their discrimination from other structurally similar mussel CS-αβ peptides.

## 2. Results and Discussion

### 2.1. Taxonomic Distribution of Mytilin-Like AMPs

The iterative screening approach of all the -omic data available for species pertaining to the family Mytilidae (see Section 3.1) allowed the identification of mytilin-like sequences in three genera, i.e., *Mytilus* Linnaeus 1758, *Perna* Philipsson 1788 and *Trichomya* Ihering 1900. While mytilins have been previously reported and studied extensively in *Mytilus* spp. during the past two decades [4,10,12,15,17,19,20,21,24], to the best of our knowledge this is the first report of the occurrence of this AMP family in other genera of marine mussels.

In order to appropriately discuss the evolutionary implications of this finding, we need to point out that the phylogenetic relationship among Mytilidae are still a matter of debate and subject to change. Indeed, recent molecular approaches have challenged the classical taxonomical classification of these organisms, revealing that morphology- and genetics-based phylogeny of mussels often show inconsistencies [28,29]. Nevertheless, the placement of some taxa is now starting to emerge with more clarity from the consensus of the most up-to-date studies. For this purpose, here we will make reference to the phylogenetic analysis recently produced by Morton and colleagues [29], which identified two major evolutionary lineages (clade A and B) within Mytilidae. At the same time, will also discuss our findings in light of the currently accepted classification, as exemplified by MolluscaBase [30].

All the three genera where mytilin sequences were identified belong to one of the two major evolutionary lineages identified by Morton and colleagues, i.e., clade A. Although *Trichomya* is currently considered as a member of the subfamily Septiferinae [30], recent molecular data support its close relatedness to *Mytilus*, suggesting that this genus should be placed within the subfamily Mytilinae [25,29]. In light of this observation, the presence of mytilins in *Trichomya* would be consistent with a shared ancestry with the *Mytilus* genus.

The phylogenetic relatedness between *Perna* and *Mytilus* has been already recognized and accepted, since both genera are considered part of the Mytilinae subfamily [30]. However, molecular evidence suggest that this genus should be reconsidered as a member of the subfamily Musculinae, closely related to the subfamily Septiferinae, which are both sister taxa to Mytilinae. Even though our screening was limited to the few mussel species with available -omic data, these findings imply that mytilins are likely be also present in other genera belonging to Musculinae and Septiferinae, such as *Musculus* Röding 1798, *Musculista* Yamamoto and Habe 1958, *Septifer* Récluz, 1848 and possibly many others that still await a revised classification.

In stark contrast, no mytilins were found in the transcriptomes available for the most basal subfamilies of clade A, including Brachidontinae, Mytiliseptiferinae and Lithophaginae clade 2 (i.e., *Lithophaga* Röding, 1798). Although the missed detection of mytilins in these taxa could be explained by the lack of expression, this hypothesis seems unlikely due to availability of a large amount of data from multiple species and tissues [28,31,32].

No mytilin-like sequences were identified in any species pertaining to the clade B from Morton et al., which includes hydrothermal vent deep-sea mussels (subfamily Bathymodiolinae), horsemussels (subfamily Modiolinae), one clade of rock-boring mussels (*Leiosolenus* Carpenter 1857, currently considered part of the Lithophaginae subfamily), the invasive mussels *Xenostrobus* B. R. Wilson 1967 and *Limnoperna* Rochebrune 1882 (both currently considered part of the Arcuatulinae subfamily). The availability of three fully sequenced genomes for this clade, i.e., *Bathymodiolus platifrons*, *Limnoperna fortunei* and *Modiolus philippinarum* [26,27], rules out the possibility of a missed detection due to lack of expression.

**Figure 2:**
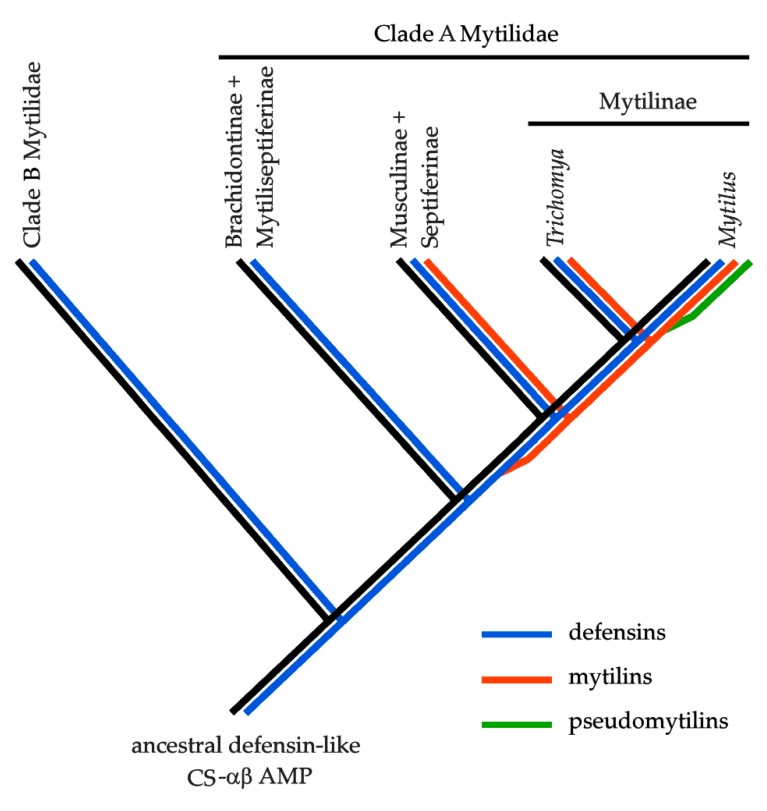
Hypothetical evolutionary scenario of the Mytilidae CS-αβ antimicrobial peptides, based on the presence/absence patterns observed in publicly available -omic data. In brief, an ancestral defensin-like gene is expected to have supported the prototypical structural scaffold for the development of all the CS-αβ AMPs currently found in extant mussel species. Mytilins are expected to have been originated by a gene duplication event that might have occurred within clade B (see Morton et al., 2019), after the split between Brachidontinae and Mytiseptiferinae from the other three subfamilies. The evolutionary origin of pseudomytilins is expected to have occurred much more recently, possibly soon after the split between the *Mytilus* and *Trichomya* lineages.

In light of these observations, and based on the assumption that all mussel CS-αβ AMPs share a common origin from a single defensin-like ancestral prototypical gene (see the Introduction), mytilins might have been originated by a gene duplication event that mostly likely occurred in clade A Mytilidae (Morton et al., 2019) after the split between the Mytilidae/Musculidae/Septiferinae and the Brachidontinae/Septiferinae clades (Figure 2). As it will be discussed in Section 2.3, pseudomytilins are expected to represent a relatively “young” *Mytilus*-specific group of mytilin-derived sequences. The evolutionary origins of myticins, which might also share the same ancestry, will be not discussed in the present work.

### 2.2. Analysis of Novel Mytilin Sequence Precursors

The different species included in the *Mytilus edulis* complex (i.e., *M. edulis*, *M. galloprovincialis*, *M. trossulus*, *M. chilensis* and *M. planulatus*) are evolutionarily closely related and locally subject to hybridization, which results in the broadly documented phenomena of genetic introgression [33]. In the present work, we chose to adopt a stringent protocol for reporting “novel” sequences, here defined as those sharing <95% primary sequence identity with those previously identified by other studies, using *M. galloprovincialis* as a reference for discussion. This approach was chosen to exclusively consider sequences, which might have evolutionary relevance, disregarding minor intra- and inter-specific variants that are expected to be found in large numbers in *Mytilus* populations.

**Figure 3:**
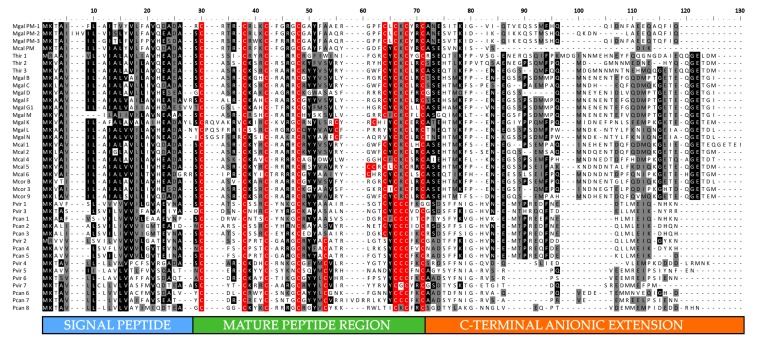
Multiple sequence alignment of full-length mytilins precursor peptides. The signal peptide, mature peptide and C-terminal anionic extension regions are highlighted. Residues conserved in >50% sequences are shaded and the cysteine residues possibly engaged in disulfide bonds are marked with a red background. Note that some nearly identical sequences have been omitted from the alignment for simplicity’s sake. PM: pseudomytilin; Mcal: *M. californianus*; Mcor: *M. coruscus*; Mgal: *M. galloprovincialis*; Pcan: *Perna canaliculus*; Pvir: *P. viridis*.

**Figure 4:**
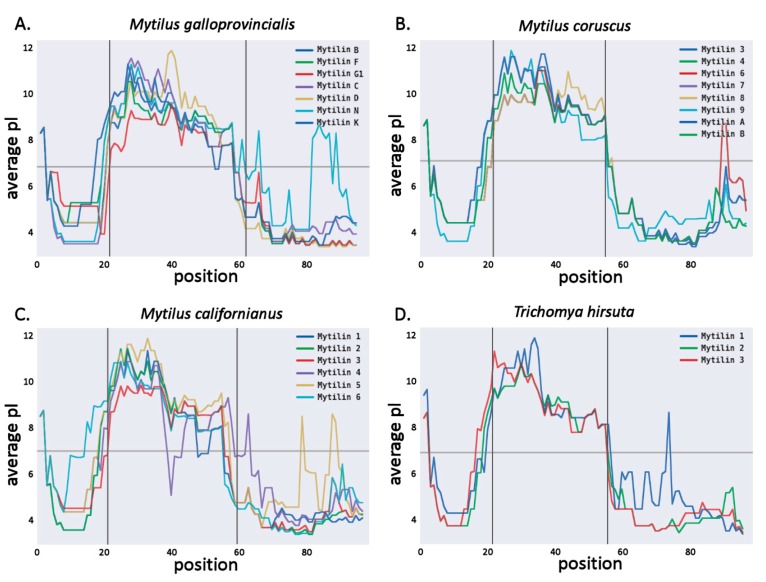
Average isoelectric point, calculated based on a sliding window of 15 aa for *M. galloprovncialis* (**panel A**), *M. coruscus* (**panel B**), *M. californianus* (**panel C**) and *T. hirsuta* (**panel D**) mytilins. The vertical bars indicate the division between the signal peptide, mature peptide and C-terminal extension regions (see Figure 3).

Overall, we identified two novel mytilin full-length sequences in the *M. galloprovincialis* transcriptome. The first one, named mytilin M, shared the highest level of homology with mytilin B (73% sequence identity). Its 101 aa long precursor peptide had a signal peptide slightly shorter than the other *M. galloprovincialis* mytilins (20 aa) and was characterized by a 34 aa long mature peptide, with the canonical 8 cysteine-disulfide array (Figure 3). The second sequence, mytilin L closely resembled mytilin N (80% sequence identity) and presented an alternative cysteine array compared with canonical mytilins, which is also found in the K isoform. The 39 aa long mytilin L mature peptide displayed an unusually long loop between C1 and C2 (7 aa), paired with an unusual position of C5 (Figure 3). The possible implications of these unique features on the topology of the C1–C5 disulfide bond will be discussed in detail in Section 2.5. Like all the other *Mytilus* mytilins, the predicted mature peptide of both MytL and MytM were highly cationic, with a +9 positive net charge (Figure 4A).

We could also identify mytilin sequences in two additional species of the *Mytilus* genus, which are not part of the *M. edulis* species complex, i.e., *M. californianus* and *M. coruscus*. Although several mytilins had been previously identified in *M. coruscus* [21,22], we can here report a novel sequence, named mytilin-9. This 101 aa long precursor sequence, processed into a 34 aa long mature peptide, shared significant similarity with other variants found in the same species (68% sequence identity, see Figure 3) and displayed a sharp shift in charge distribution between the cationic mature peptide region and the anionic C-terminal extension (Figure 4B).

A total of six different mytilin sequences were identified in *M. californianus*, representing the first report of this AMP family in the species. In general, these sequences showed a higher level of similarity with the mytilins from *M. coruscus* (in the range of 78–87%) than with those found in the *M. edulis* complex, consistently with the phylogenetic placement of the species [25]. The length of the precursor and mature was very similar (102–106 aa and 34–35 aa, respectively), and all sequences displayed a canonical cysteine array (Figure 3). As previously described for the *M. galloprovincialis* mytilins, a sharp shift in the amino acidic composition of the precursor protein was visible in correspondence with the predicted mature peptide cleavage site, highlighting a clear distinction between the positively charged mature region and the negatively charged C-terminal extension (Figure 4C).

We were also able to identify a few additional sequences closely related with pseudomytilin [10]. In detail, pseudomytilins were found in *M. galloprovincialis* (two sequences in addition to the one previously reported) and *M. californianus* (one sequence). While the *M. californianus* sequence displayed an apparently truncated C-terminal extension, the two *M. galloprovincialis* precursor peptides had a similar precursor and mature peptide length (92–95 aa and 43 a, respectively, see Figure 3). Compared with the other mytilin-like sequences described so far, the high positive net charge of the mature peptide (+8/+10) was not matched by the anionic C-terminal extension, which only displayed a weak negative charge.

We identified three mytilin sequences in *T. hirsuta*, with a precursor length of 102–106 aa and 32–34 aa long predicted mature peptides with canonical cysteine arrays (Figure 3), similar to those of *Mytilus* (i.e., 46–69%, see Figure 3). The mature peptides displayed a positive total net charge (+7/+10), which was counterbalanced by the negative charge of the anionic C-terminal extension (−6/−8; Figure 4D).

A much higher number of mytilin-like sequences were found in two distinct species of the *Perna* genus, i.e., the Asian and New Zealand green mussels *Perna viridis* (seven variants) and *Perna canaliculus* (eight variants). All the precursor proteins were 83–90 aa long, with a 33–37 aa long predicted mature peptide and a C-terminal extension that did not bear a remarkable primary sequence similarity with those of *Mytilus* and *Trichomya* (Figure 3). Although, like the other mytilins, those found in *Perna* usually displayed a charge separation between the mature peptide and the C-terminal extension, the cationicity of the mature peptides was of minor entity, with some extreme cases showing nearly neutral charge at pH 7. In detail, the two regions averaged a net charge equal to +4.33 (ranging from 0 to +11) and −3.4 (ranging from 0 to −7), respectively.

The most peculiar feature of *Perna* mytilins was that 13 of the 15 sequences identified had an extra cysteine residue placed between the C6 and C7 of the canonical array. This resulted in the presence of an odd number of cysteine residues and in the creation of an unusual “CCC” motif, whose possible functional implications will be discussed in Section 2.5. At the same time, many *Perna* mytilins were characterized by an alternative disulfide array, similar to the one found in *M. galloprovincialis* mytilin K, L and N, due to variation in the position of C5 (Figure 3). It needs to be further noted that one mytilins from *P. viridis* lacked the C8 of the canonical array.

All the novel sequences identified in the present study have been deposited in GenBank under the accession IDs: MN883571–MN883600. The mature peptide primary sequences are listed in Table 1.

### 2.3. Molecular Evolution of Mytilins

**Figure 5:**
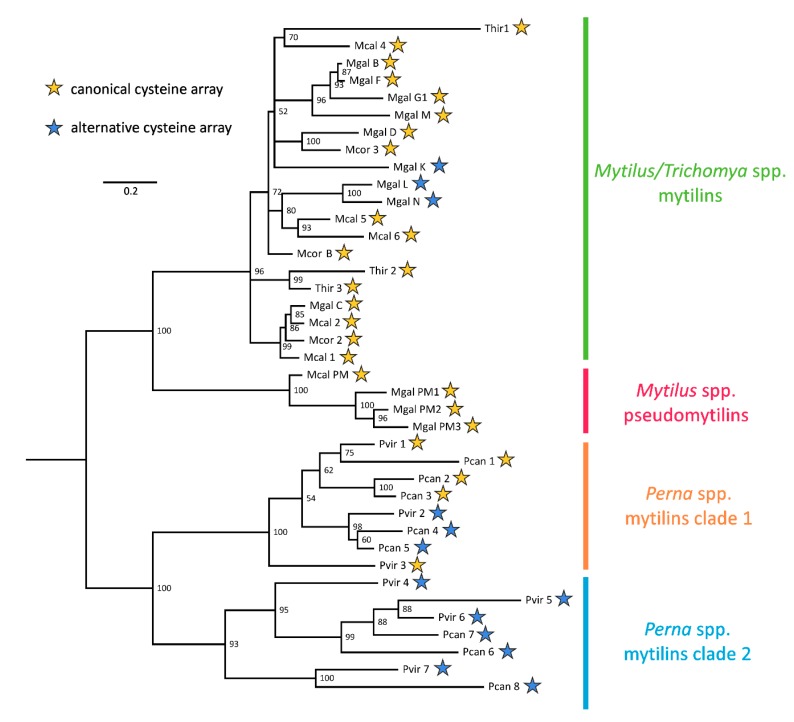
Bayesian tree built from all mytilin-like sequences, based on a JTT + I + G model of molecular evolution (see Section 3.4). Nodes below 50% support were collapsed. PM: pseudomytilin; Mcal: *M. californianus*; Mcor: *M. coruscus*; Mgal: *M. galloprovincialis*; Pcan: *P. canaliculus*; Pvir: *P. viridis*. For a definition of the canonical and alternative cysteine array, see Section 2.2.

The JTT + I + G4 rate heterogeneity model of molecular evolution (i.e., the one proposed by Jones, Taylor and Thornton [34], with a proportion of invariant sites and Gamma-distributed rate of variation across sites) was identified as the best fitting one for mytilins. The Bayesian tree (Figure 5) showed a sharp and highly supported separation between the mytilins from *Mytilus* and *Thricomya* spp. and those from *Perna* spp. (100% posterior probability support). Based on the hypothesized derivation of mytilins from an ancestral defensin-like gene (see Figure 2), this hypothetical sequence would be expected to occupy a basal position in the tree.

The mytilins from *Perna* displayed a remarkable diversification, with a subdivision between two divergent clades shared by both *P. viridis* and *P. canaliculus*. This subdivision, supported by a highly significant posterior probability values (93%, see Figure 5), mirrored the visible primary sequence divergence of the C-terminal extension (see Figure 3), but did not match the presence of a canonical or alternative cysteine array (see Section 2.2). Overall, the phylogenetic tree indicates that the ancestral *Perna* mytilin gene might have undergone an initial duplication very early along the evolution of the genus, and further implies that the mytilin CS-αβ structural scaffold allows a large degree of freedom concerning the position of C5, which has been independently modified in sequences belonging to both the *Perna* and the *Mytilus* lineages, generating an alternative disulfide array (Figure 3 and Figure 5), whose implications will be discussed in Section 2.5.

All the *Mytilus* and *Trichomya* spp. mytilins were placed, intermixed, in a well supported monophyletic clade (96% posterior probability, see Figure 5), and overall displayed a lower level of molecular diversity compared with *Perna*. The sequences from the different *Mytilus* species were often mixed, consistently with their hypothesized recent origin by gene duplication, as well as with the proven dispensable nature of some *M. galloprovincialis* mytilin genes [25]. The Bayesian tree also indicated the derivation of pseudomytilins from canonical mytilins, as suggested by their placement in a divergent highly supported early-branching clade (100% posterior probability, see Figure 5). Although no pseudomytilins have been identified in *T. hirsuta*, the topology of the tree suggests that this absence might be simply due to a lack of expression in the few transcriptomic datasets available for this species (Figure 2).

The multiple sequence alignment of all mytilins (Figure 3) clearly identified the extreme sequence divergence of the C-terminal anionic extension region as a key factor in determining the placement of the precursor peptides in the four evolutionary lineages evidenced in Figure 5. This observation may be consistent with the “exon shuffling” hypothesis, previously postulated by other authors for the genes encoding defensin-like antimicrobial peptides [35]. This evolutionary model would imply the independent recruitment along evolution by the central CS-αβ module of different and unrelated upstream or downstream sequences. However, we argued that alternative scenarios, such as more relaxed selective constraints acting on the C-terminal region, might have produced similar results. The future analysis of additional genomes from Mytilidae is certainly expected to clarify the possible impact of exon shuffling on mytilin gene evolution.

### 2.4. Building of a Mytilin-Specific Hidden Markov Model

Here we proposed a new mytilin-specific HMM, built by exploiting the improved knowledge derived from the discovery of the novel sequences presented in the present work, which might provide a support for the future identification of additional mytilin sequences in the genomes, transcriptomes and proteomes of other mussel species where mytilins are expected to be found (see Section 2.1).

The construction of the HMM (available in Supplementary File S1) was based on 39 unique sequences, selected with a clustering approach (see Section 3.3). This allowed the removal of redundant, nearly identical sequences, enabling a balanced representation of the remarkable sequence variation shown in Figure 3 and Figure 5.

The test of the HMM model revealed a high level of sensitivity, as it was able to identify with significant e-values (i.e., lower than 1 × 10^−10^) all the mytilin peptides described in the present work (Supplementary Table S1). The specificity of the method was also tested by screening the full set of sequences analyzed in this work, which included previously known sequences of other mussel CS-αβ AMPs. We observed that this method produced a few false positives, mostly belonging to the myticin AMP family, even though the e-values associated with such matches were generally close to the arbitrary threshold of significance (0.05). Due to the evolutionary and structural link between the two *Mytilus* AMP families (see the Introduction), this result was expected. We suggest that the HMM search for mytilin sequences should be always paired with a parallel screening performed with a myticin-specific HMM. Preliminary tests indicate that this strategy would allow a reliable discrimination between the two AMP families (Supplementary Table S1).

### 2.5. Structural Prediction

Our threading approach succeeded in building reliable three-dimensional structural models for nearly all the primary sequence analyzed, with the only exception of two sequences from *Perna* spp., whose models were poorly supported (Supplementary Table S2). All the predicted models displayed a very similar structure and shared the CS-αβ backbone, with the only exception of pseudomytilins, where the two β-strands were modeled as coils. This result may be consistent with the inaccuracies typically linked with β-sheet prediction [36], and therefore it should not be considered as an indication that pseudomytilins are lacking these secondary structures.

**Figure 6:**
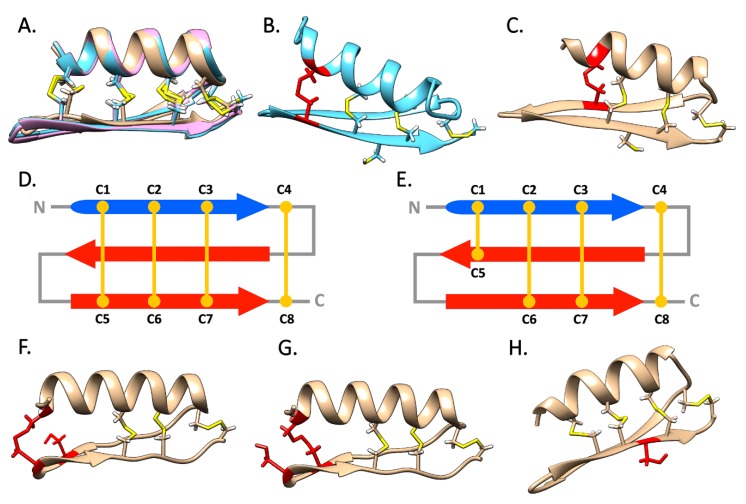
**Panel A**: super-imposition of the structural models obtained for *T. hirsuta* mytilin 1, 2 and 3. **Panels B** and **C**: structural models of *Perna* mytilins characterized by the presence of a canonical and an alternative cysteine array, respectively; the variable position of the C1–C5 disulfide bond is highlighted in red. **Panels D** and **E** schematic organization of the disulfide bond topology of mytilins with a canonical and alternative cysteine array, respectively. **Panels F** and **G**: alternative structural variants for *M. galloprovincialis* mytilin K, with two different sterically allowed C1–C5 disulfide bonds, highlighted in red. **Panel H**: structural model of a *Perna* defensin, with the side chain of the central cysteine residue of the CCC motif pointing outwards, highlighted in red.

Despite their high primary sequence divergence (Figure 3), the mytilins from *Perna* and *Mytilus/Trichomya* models were almost overlapping (see Figure 6A as an example). However, the variable position of C5 in most *Perna* and some *Mytilus* mytilins (see Section 2.2) had a significant effect on the topology of the disulfide array, with important structural (and possibly also functional) implications. For example, the predicted models from *Perna* could be divided between two well-distinct structural subgroups, which were fully consistent with the two alternative arrays highlighted in Section 2.2 (see Figure 5). In detail, the first subgroup included all the *Perna* mytilins characterized by the canonical cysteine array: in this case, the C1–C5 disulfide bond was predicted to connect the α-helix with the second β-strand of the CS-αβ fold (Figure 6B), perfectly matching the disulfide connectivity previously described for *M. galloprovincialis* mytilin B [11] (Figure 6D). On the other hand, the second subgroup comprised all the *Perna* mytilins characterized by the presence of an alternative cysteine array. In these sequences, the C1–C5 disulfide bond was predicted to link the α-helix with the first β-strand, where C5 was located (Figure 6C,E). Strikingly, the C1–C5 disulfide bond of the three *M. galloprovincialis* sequences with an alternative cysteine array (mytilin L, K and N, see Figure 3 and Figure 5) was also placed in a similar position, right before the loop connecting the two β sheets. Curiously, mytilin K displays nine cysteines, and two of such residues are located close to the expected position of C5 (see Figure 3). Structural modeling shows that both cysteine residues would be sterically allowed to form a disulfide bond with C1, potentially producing two alternative structural isoforms (Figure 6F,G).

The CCC motif found in most mytilins from *Perna* represents another structural peculiarity. As predicted by the alternate disposition of R groups in amino acid polymers, the first and the third residues of the triad are likely to be involved in the formation of the C2–C6 and C3–C7 disulfide bonds, whereas the side chain of the second cysteine residue is expected to point outwards (Figure 6H). Altogether, the accessibility of this side chain on the molecular surface, its unlikely involvement in the formation of intra-molecular disulfide bonds and the conservation of this residue in 13 out of the 15 *Perna* mytilins identified in this study, suggest that it could be engaged in an inter-molecular disulfide bond, resulting in the formation of homo- or heterodimers.

Although no bivalve AMP has ever been reported to exert its antimicrobial activity as a dimer, a handful animal AMPs have been identified as active dimers, either stabilized by inter-molecular disulfide bonds, or by weak noncovalent interactions [37,38,39,40]. Clearly, while these observations pave the way to new hypotheses concerning the mode of action of mussel CS-αβ AMPs, the possibility that *Perna* mytilins may act as dimers will require experimental validation through the isolation of the native peptides from the hemocytes.

**Figure 7:**
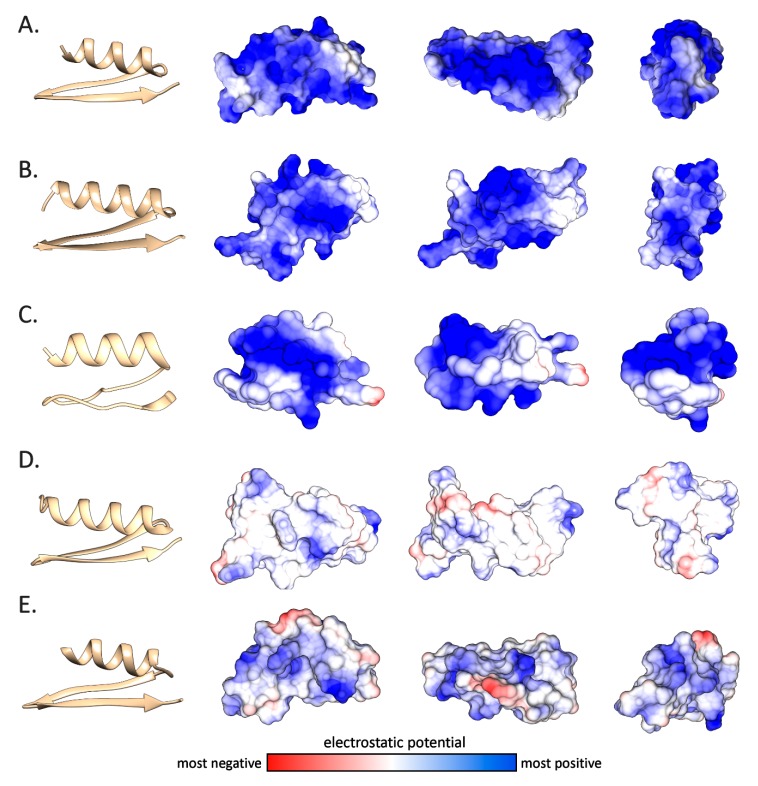
Ribbon structural models and electrostatic potential of five mytilins, displayed on the molecular surface of the molecules, rotated on the three main axes. **Panel A**: *T. hirsuta* mytilin 2. **Panel B**: *M. galloprovincialis* mytilin L. **Panel C**: *M. galloprovincialis* pseudomytilin 2. **Panel D**: *P. canaliculus* mytilin 1. **Panel E**: *P. viridis* mytilin 5.

The structural predictions also allowed us to investigate more in detail the electrostatic potential distribution on the molecular surface of the mytilin mature peptides (Figure 7). The cationic nature of the canonical mytilins from *Mytilus* and *Trichomya* spp. (panels A and B), as well as for pseudomytilins (panel C) was well-evident, with a nearly uniform distribution of positive charges across the entire molecular surface, which was in line with the isoelectric point plots obtained from the analysis of primary sequence data (Table 1 and Figure 4). On the other hand, as mentioned in Section 2.2, *Perna* mytilins generally displayed a weaker positive net charge, to the point that a few mature peptides showed a nearly neutral charge (Table 1). This was quite notable, for example, in the case of *P. canaliculus* mytilin 1 (Figure 7D). It can be expected that this feature may possibly result in a lower binding affinity towards the negatively charged bacterial cell membranes, with a consequent decreased antimicrobial potential. Whether this unusual electrostatic potential distribution is linked with the acquisition of alternative cytokine-like functions, as in the case of myticins, remains to be investigated [7].

## 3. Materials and Methods

### 3.1. Identification of Mytilin Sequences

Sequencing reads and—where available—reference transcriptomes from the studied species (Bathymodiolus azoricus, Bathymodiolus childressi, Bathymodiolus manusensis, B. platifrons, Bathymodiolus puteoserpentis, Geukensia demissa, Lithophaga lithophaga, Modiolus kurilensis, Modiolus modiolus, M. philippinarum, Mytilisepta virgata, M. californianus, M. chilensis, M. coruscus, M. edulis, M. galloprovincialis, M. planulatus, M. trossolus, P. canaliculus, P. viridis, Perumytilus purpuratus and T. hirsuta) were downloaded from NCBI SRA and TSA databases, as reported in (Supplementary Table S3).

The transcriptome assemblies for the collected reads were performed separately for each target species, using two distinct methods, i.e., Trinity v2.8.6 [41] and the CLC Genomics Workbench v12.0.1 (https://www.qiagenbioinformatics.com/), in order to investigate and solve potential misassembly issues through the comparison between the two methodologies. Moreover, the fully sequenced and annotated genomes of *L. fortunei* [27], *M. philippinarum* and *B. platifrons* [26] were downloaded and included in the workflow.

All transcriptomes and genomes were used to generate BLAST databases. Known mytilin and pseudomytilin sequences from *M. coruscus* [21] and *M. galloprovincialis* [4,10,42] were used to build a seed sequence list that was used as a query in a tBLASTn search against such databases, with an e-value threshold of 1 × 10^−3^. The resulting transcriptome hits were then checked for the presence of complete open reading frames (ORFs), which were translated and aligned with the sequences included in the seed list, along with mussel defensins and myticins, in order to (i) confirm their classification within the mytilin family and (ii) allow their discrimination from the other two mussel CS-αβ AMP families. A similar process was used for genome hits, but in this case existing exon annotations were used to predict ORFs and to obtain the putative encoded proteins. Each verified hit was then added to the seed sequence list and this process was re-iterated until no new significant hits could be found in the screened genomes and transcriptomes.

### 3.2. Primary Sequence Analysis

All the detected mytilin-like sequences were analyzed with SignalP v4.1 [43] to identify the signal peptide cleavage site and aligned using MUSCLE [44]. This process, through the alignment with *M. galloprovincialis* mytilin B, whose precursor structure had been previously characterized [17], also enabled the identification of the putative cleavage site for the C-terminal anionic extension. The charge distribution of each peptide was analyzed through the calculation of the average isoelectric point based on a sliding window of 15 amino acids.

### 3.3. Construction of a Mytilin-Specific Hidden Markov Model

The complexity of the full set of mytilin-like sequences was reduced by removing redundant data. This was achieved by building a pairwise blosum 62 [45] distance graph of all sequences and by iteratively removing the neighbor sequences displaying a pairwise distance lower than the arbitrary threshold of 0.1 (set based on preliminary trials), as described in the algorithm 2 from Hobohm and colleagues [46]. The resulting multiple sequence alignment was used to build a HMM using hmmer v3.3 [47], which was subsequently tested on a set of known mussel AMPs to evaluate its performance, both in terms of sensibility and specificity.

### 3.4. Phylogenetic Analysis

The same multiple sequence alignment file used for the generation of the HMM was used to perform a phylogenetic analysis. Briefly, the best-fitting molecular model of evolution for this sequence set was evaluated with ModelTest-ng v.1.6 [48], based on the corrected Akaike information criterion. Bayesian inference analysis was run with a Monte Carlo Markov Chain approach with MrBayes v3.2.7a [49,50]. Two independent analyses with four chains each were run in parallel for 2 million generations, i.e., until the estimates of all the parameters of the model reached convergence. This was assessed with Tracer v1.7.1 [51], by ensuring that the effective sample size for all parameters reached a value >200. The resulting phylogenetic tree, constructed as a 50% majority-rule consensus tree, was graphically rendered using FigTree v1.4, collapsing all nodes with posterior probability support <0.5 [52].

### 3.5. Structural Prediction

The three-dimensional structural prediction of selected mature mytilin peptides was performed with a threading/fold-recognition approach using I-TASSER v5.1 [53,54,55,56]. In detail, the N-terminal end of the mature peptides was predicted based on the detection of the signal peptide cleavage site with SignalP v4.1 [43], and the putative C-terminal end of the mature peptide was identified based on the alignment with the known mature peptides previously described by other authors [17]. Structural modeling was performed for all the novel sequences identified in this study. The reliability of the structural models obtained was evaluated with the *c-score* and the *z-score* metrics, calculated by I-TASSER and ProSA-web, respectively [57,58]. The graphical rendering and optimization of the 3D structures were performed with USCF Chimera v1.14 [59]. This process was carried out by fixing the bonds with respect with the expected disulfide connectivity and by performing energy minimization, followed by re-evaluation of the resulting 3D structure.

## 4. Conclusions

This study reported for the first time the presence of mytilins outside the *Mytilus* Linnaeus 1798 genus, extending the taxonomical range of distribution to *Perna* Philipsson 1788 and *Trichomya* Ihering 1900. Our results further suggest that several additional mytilins might be found in other still unexplored mussel species belonging to the Mytilinae and Musculinae subfamilies, and we built a HMM that should streamline their detection in the future from the output of genome or transcriptome sequencing efforts.

Altogether, we brought further insights into the evolution of mussel CS-αβ AMPs, revealing that: (i) the molecular diversity of these defense molecules is higher than previously reported and (ii) the mussel CS-αβ structural scaffold allows a significant degree of structural flexibility, as revealed by the shifting position of C5 and the corresponding modification in the topology of the C1–C5 disulfide bond, which may link the alpha helix either with the first or with the second beta strand of the CS-αβ fold.

Since our investigation has been restricted to the very few genera out of the 63 currently included in the family Mytilidae (i.e., only those with available -omic information), our observations suggest that, along with the discovery of novel AMPs in Mytilidae, the nomenclature scheme of these molecules might require some modification, consistently with the previously reported widespread inconsistencies that affect the nomenclature of CS-αβ peptides in scientific literature [60]. As a matter of fact, the current distinction between mytilins, myticins and defensins is likely to become more and more unclear and AMPs with mixed features are likely to be uncovered.

## Figures and Tables

**Table 1:** List of the novel mytilin sequences identified in this study. Cysteine residues are highlighted in bold. PM: pseudomytilin; Mcal: *M. californianus*; Mcor: *M. coruscus*; Mgal: *M. galloprovincialis*; Pcan: *P. canaliculus*; Pvir: *P. viridis*.

Sequence Name	Mature Peptide Sequence	Length (aa)	Net Charge
Mcal mytilin 1	SCASRCKYRCRARRCRYYVSVRYGWFCYCRCLHC	34	+9
Mcal mytilin 2	SCASRCKSRCRARRCKYYVSVRYGWFCYCRCLRC	34	+10
Mcal mytilin 3	SCALLCKAHCRARRCGYYVSVFYHGRCYCRCLRC	34	+7
Mcal mytilin 4	SCASKCKAVCRRRRCAGYDWVLWGGHCFCKCSRC	34	+7
Mcal mytilin 5	SCASRCKYRCRRRRCRSYVAVRYCCRCLCKCRRC	34	+13
Mcal mytilin 6	SCIPRCKYICTRRRRCGYYAAIYYCHRCYCKCLSC	35	+8
Mcal PM	SCRTRCRFKCFGRGCGAYFAAQYGDFCYCKCYRC	34	+6
Mcor mytilin 9	SCASRCKSRCRARRCRYYVAVRYGWFCYCRCLRC	34	+10
Mgal mytilin L	YCPQSFRRICSSRCRGRGCQYYVAVCFPRRYYCKCLRC	38	+9
Mgal mytilin M	SCASRCRSHCRARRCHYSKSVLVGRRCFCKCFLC	34	+9
Mgal PM 2	SCRTRCRLKCFGRGCGAYFAAQRGPFCLCKCYRC	34	+8
Mgal PM 3	SCRSRCRWKCFRRRCGAYFAAQRGPFCLCKCYRC	34	+10
Pcan mytilin 1	SCDRWCNTSCYNKGCRYYAASVSDGRCFCCCITC	34	+2
Pcan mytilin 2	NCARSCSSRCYHRNCKAYASVYRNETCYCCCIDC	34	+3
Pcan mytilin 3	SCATSCSSRCEYRKCEDYASAIRDGKCYCCCIKC	34	+2
Pcan mytilin 4	NCFSCPSTCARRGCRYFACATRLRKSYCCCFVC	33	+6
Pcan mytilin 5	SCFSCPRTCGARGCRYYACATRFGTSYCCCFKC	33	+5
Pcan mytilin 6	YCDLCRWYCSNKGCAYYLCGNKFGNNYCCCFKC	33	+3
Pcan mytilin 7	YCDRCREYCSNTGCGYYMCVRRIVDRRLKYYCCCFKC	37	+5
Pcan mytilin 8	GCGGCKYKCRRRGCRGYVCYKKRWLTICKCFRC	33	+11
Pvir mytilin 1	SCATSCSSRCYNKGCKYYAAAIRSGTCYCCCFKG	34	+5
Pvir mytilin 2	SCSSCPRTCGARGCRYYACATRLGTSYCCCFKC	33	+5
Pvir mytilin 3	DCDSNCNHRCYYRGCKAYASALNNGTCYCCCVDC	43	0
Pvir mytilin 4	SCARCKDHCRNKGCGFYMCVLRYGTYYCCCFKC	33	+5
Pvir mytilin 5	NCERCKYYCSYKNCSQYMCVRHNANDYCCCFNC	33	+2
Pvir mytilin 6	ACDRCKAYCTIKGCGYYLCVHRFPSYYCCCFKC	33	+4
Pvir mytilin 7	SCYTCKRRCAARGCRYYLCVIRYYRVYCGCYRC	33	+8
Thir mytilin 1	SCSSICRYRCRRCRGFIWINIFGRCYCKCYGC	32	+7
Thir mytilin 2	SCASSCKSRCRSRGCKYFVSVRYRYHCYCKCLRC	34	+9
Thir mytilin 3	SCASRCKSRCRARRCKYYVSVRYGWFCYCKCLRC	34	+10

## Supplementary Materials

The following are available online at https://www.mdpi.com/2079-6382/9/1/37/s1, File S1: mytilin-specific hidden Markov model, Table S1: performance of the hidden Markov model against mytilin and myticin sequences, Table S2: *c-score* and *z-score* of the structural models predicted by I-TASSER; Table S3: list of SRA accession IDs used in the present work.
